# Monitoring activity in neural circuits with genetically encoded indicators

**DOI:** 10.3389/fnmol.2014.00097

**Published:** 2014-12-05

**Authors:** Gerard J. Broussard, Ruqiang Liang, Lin Tian

**Affiliations:** ^1^Department of Biochemistry and Molecular Medicine, University of California DavisDavis, CA, USA; ^2^Neuroscience Graduate Group, University of California DavisDavis, CA, USA

**Keywords:** *in vivo* imaging, mammalian and rodent brain, neural activity, genetically encoded sensors, calcium sensor, voltage sensor, synaptic activity reporter

## Abstract

Recent developments in genetically encoded indicators of neural activity (GINAs) have greatly advanced the field of systems neuroscience. As they are encoded by DNA, GINAs can be targeted to genetically defined cellular populations. Combined with fluorescence microscopy, most notably multi-photon imaging, GINAs allow chronic simultaneous optical recordings from large populations of neurons or glial cells in awake, behaving mammals, particularly rodents. This large-scale recording of neural activity at multiple temporal and spatial scales has greatly advanced our understanding of the dynamics of neural circuitry underlying behavior—a critical first step toward understanding the complexities of brain function, such as sensorimotor integration and learning. Here, we summarize the recent development and applications of the major classes of GINAs. In particular, we take an in-depth look at the design of available GINA families with a particular focus on genetically encoded calcium indicators (GCaMPs), sensors probing synaptic activity, and genetically encoded voltage indicators. Using the family of the GCaMP as an example, we review established sensor optimization pipelines. We also discuss practical considerations for end users of GINAs about experimental methods including approaches for gene delivery, imaging system requirements, and data analysis techniques. With the growing toolbox of GINAs and with new microscopy techniques pushing beyond their current limits, the age of light can finally achieve the goal of broad and dense sampling of neuronal activity across time and brain structures to obtain a dynamic picture of brain function.

## INTRODUCTION

Within the mammalian brain, neuronal and glial cells communicate at spatial and temporal scales spanning orders of magnitude. One of the fundamental challenges with which modern neuroscience is currently grappling is the development of tools that can record this communication as it occurs at the relevant scales. Furthermore, it is desirable that these tools should be deployable in living animal models. Such probes will aid the study of neural communication within the context of phenomena such as experience dependent plasticity, sensorimotor integration, learning, and memory.

An extensive set of tools for studying brain function *in vivo* currently exists, and each of these possesses its own set of strengths and weaknesses. For example, recordings of intracellular voltage or transmembrane current can be made using patch clamp (Liu et al., 2009; Crochet et al., 2011) or extracellular potential recordings can be achieved with fine-tipped metallic electrodes (Hubel and Wiesel, 1959; Reid et al., 1997; Mante et al., 2013). These methods provide an exquisitely detailed temporal signal, but are limited by the number of cells that can be recorded simultaneously. Simultaneous neural recordings with near-cellular resolution can be achieved using multiple electrode arrays (Buzsaki, 2004). However, this technique cannot precisely localize cell position and yields limited information about cell types. Optical imaging of bolus loaded AM ester dyes allows recordings at high spatial resolution from many cells simultaneously (Stosiek et al., 2003; Helmchen and Denk, 2005). But this technique results in high background due to residual fluorescence in the extracellular space as well as the lack of genetic control (Stosiek et al., 2003). A lack of genetic control can in particular lead to increased fluorescent background due to unspecific staining of non-neuronal cells as well as neurons not pertinent to a given study. In addition, small molecule-based dyes are not compatible with chronic imaging (Aramuni and Griesbeck, 2013). Both small-molecule dyes and electrode methods are highly invasive and can negatively impact the health of recorded cells (Polikov et al., 2005; Dombeck et al., 2010) making chronic recordings from the same cell population difficult.

Genetically encoded indicators combined with modern microscopy (such as multi-photon microscopy), can potentially overcome these challenges to allow non-invasive, ultrasensitive and chronic measurement at specific synapses and within or across circuit elements in behaving animals. A large array of protein based indicators has been created to monitor neurotransmission, synaptic spillover, excitable membrane potential, calcium dynamics, vesicle trafficking, receptor mobilization and other essential biochemical events related to neural activity. A subset of these—detecting changes in intracellular calcium concentration, synaptic signaling events, and changes in membrane potential—have been successfully deployed for the imaging of action potentials (APs) *in vivo*. Here, we refer to this group of probes as genetically encoded indicators of neural activity (GINAs). For a more exhaustive review of available fluorescent sensors of cellular activity, please see VanEngelenburg and Palmer (2008), Ibraheem and Campbell (2010). Application of GINAs have facilitated large-scale recording of neural activity in genetically identified populations over multiple spatial and time scales in living neurons *in vitro*, *ex vivo*, and *in vivo*.

Here we review advances in design and engineering of GINAs. We discuss various properties of genetically encoded calcium indicators (GECIs) and their optimization for improved detection of single and multiple APs in *in vivo* imaging. In particular, as a case study, we present protein engineering efforts to incrementally improve intrinsic properties of the GCaMP family to match with challenging signal to noise ratio (SNR) in *in vivo* imaging. We further discuss technologies developed to aid *in vivo* imaging in the rodent brain. Finally, we briefly summarize recent findings based on the strength of GINAs as a toolbox for analyzing neural circuit function.

## DESIGN OF GENETICALLY ENCODED INDICATORS OF NEURAL ACTIVITY

**FIGURE 1:**
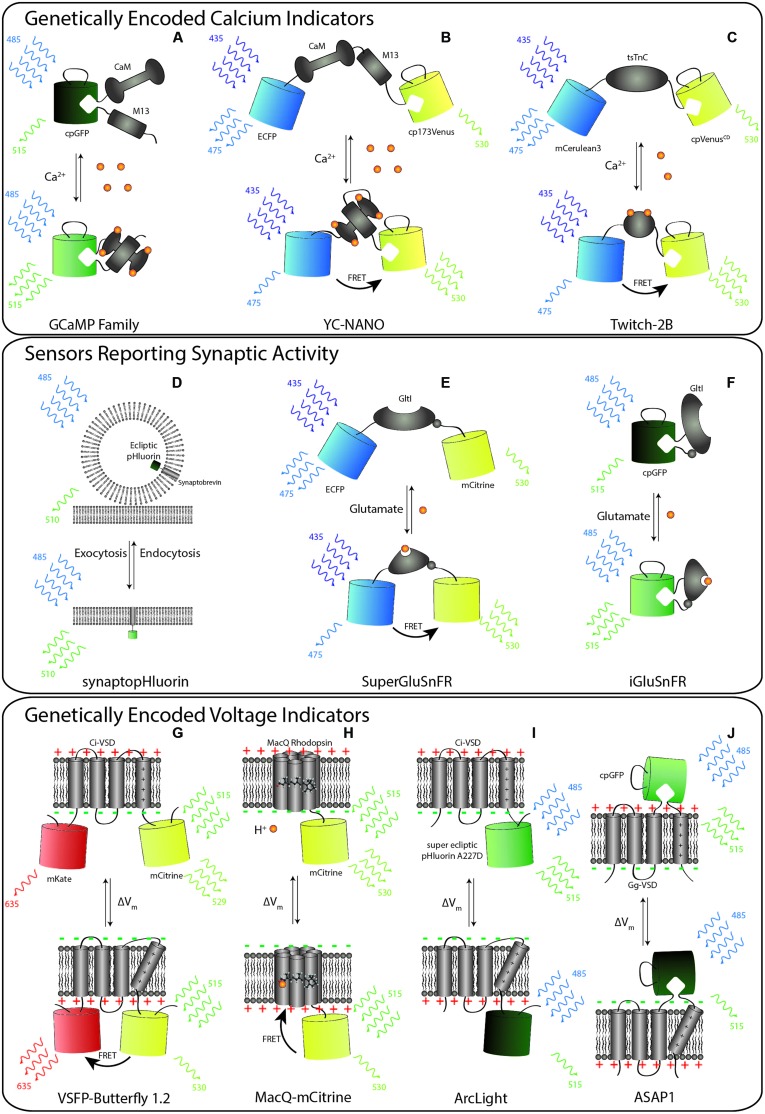
**Schematic representation of selected members of genetically encoded indicators of neural activity (GINA). (A–C)** Calcium indicators. **(A)** GCaMP6 consists of circularly permuted green fluorescent protein (cpGFP) inserted between calmodulin (CaM) and an M13 peptide. Upon calcium binding, conformational changes in the CaM–M13 complex induce fluorescence changes in the circularly permuted enhanced green fluorescent protein (cpEGFP). **(B)** YC-Nano contains CaM–M13 complex sandwiched between the donor FP (ECFP) to the acceptor FP (cp173Venus). Upon calcium binding, conformational changes of CaM–M13 complex increases Förster resonance energy transfer (FRET) between the FP pair. The result is an increase in the ratio of the fluorescence in the yellow to blue channels. **(C)** Likewise, Twitch-2B increases FRET upon calcium being bound by its calcium sensitive domain, tsTnC. **(D–F)** GINAs reporting synaptic activity. **(D)** SynaptopHluorin consists of a ecliptic pHluorin FP fused to the C-terminus of the vesicular protein. Vesicular release places the FP in the low pH environment of the extracellular space, which leads to the deprotonation of the fluorophore and increases pHluorin fluorescence. **(E)** SuperGluSnFR is a linear fusion of CFP, the *Escherichia coli* glutamate binding domain, GltI, and mCitrine. Upon glutamate binding, the conformational changes of GltI result in FRET between CFP to mCitrine. **(F)** iGluSnFR contains a cpGFP fused to the glutamate binding domain. The conformation changes of Gltl induced upon glutamate binding results in deprotonation and increased fluorescence of cpGFP. **(G–J)** Genetically encoded voltage indicators. **(G)** Upon membrane depolarization, the voltage-sensing domain of Ci-VSP enters its activated state. The FPs of voltage sensitive fluorescent protein (VSFP)-Butterfly 1.2 are thus drawn into close proximity to one another, increasing FRET. **(H)** The opsin-based MacQ-mCitrine reduces fluorescence output upon membrane depolarization. This effect is driven by enhanced FRET from the mCitrine FP to the weakly fluorescent retinal caused by a shift in the cofactor absorption spectrum upon protonation of the Schiff base. **(I)** ArcLight consists of Ci-VSD and super ecliptic pHluorin carrying the point mutation A227D. Depolarization of the membrane results in a decrease in the fluorescence output of the pHluorin, but the mechanism of this change remains elusive. **(J)** ASAP-1 contains cpGFP fused into an extracellular linker (S3–S4 linker) of the Gag-VSP voltage sensitive domain. Depolarization quenches the fluorescence of the cpGFP.

The first protein used to detect functionally relevant changes within a cell was an aequorin protein which was purified from jellyfish (Shimomura et al., 1962) and introduced exogenously to muscle fibers of a barnacle to sense changes to intracellular calcium that occur during fiber contraction (Ashley and Ridgway, 1968). The era of the modern GINAs, however, began in earnest with the introduction of biosensors based on fluorescent proteins (FPs) which were able to detect changes to intracellular calcium levels (Miyawaki et al., 1997; Romoser et al., 1997), membrane voltage (Siegel and Isacoff, 1997) and synaptic vesicle secretion (Miesenbock et al., 1998). A guide to the general design of several GINA families is presented in **Figure 1**.

Genetically encoded indicators of neural activity typically consist of an analyte-binding or sensing domain and a reporter element which is based on either a single FP or two FPs. In the case of single FP GINAs, changes in the cellular environment detected by the analyte-binding or sensing domain result in changes in the chromophore environment of the FP leading to an increment or decrement of fluorescence intensity (Siegel and Isacoff, 1997; Baird et al., 1999; Nakai et al., 2001). For example, as illustrated diagrammatically in **Figure 1A**, in the scaffold of GCaMPs, calmodulin (CaM) binds the M13 peptide from myosin light chain kinase in the presence of calcium; this coupling reverses when calcium is absent (Nakai et al., 2001). The sensor domain transduces conformational changes of analyte binding to a change in the fluorescence intensity through its coupling with the reporter FP(s). Additionally, some single FP-based GINAs contain FPs in which fluorescence intensity is environmentally sensitive (e.g., pH sensitive FPs; **Figure 1D**; Miesenbock et al., 1998). In the case of two FP based sensors, the conformational changes in the analyte-binding or sensing domain lead to Förster resonance energy transfer (FRET) between two FPs with overlapping excitation and emission spectra. For example, the Cameleon family contains both M13 and CaM in between a blue/green or cyan/yellow FP pairs as shown for Yellow Cameleon-Nano (YC-Nano; **Figure 1B**). Upon calcium binding, the relative distance and orientation of the FPs are altered, resulting in a change in the non-radiative transfer of energy between the donor and acceptor chromophores.

Single-FP based GINAs are prone to motion artifacts, which can be corrected by algorithms (e.g., Dombeck et al., 2007) and using a reference fluorophore. On the other hand, the FRET based GINAs, by virtue of ratiometric imaging can cancel the motion-related artifacts (Wallace et al., 2008; Lütcke et al., 2010) and are thus highly suitable for imaging neuronal activity in freely moving animals. However, the SNR of FRET-based probes are lower than their single FP counterparts in probing higher numbers of APs (see, e.g., Tian et al., 2009). Single-FP based probes also preserve spectral bandwidth for applications in multiplex imaging and optogenetics. Although the currently used FRET sensors can be coupled with red-shifted FPs and optogenetic tools, the broad emission and excitation spectra of FRET sensors reduce some flexibility.

Relative to other methods for recording neural signals, GINAs possess several advantages. First, they can be targeted to genetically defined cell populations of interest. As such, they can be introduced via minimally invasive interventions such as viral mediated gene delivery or transgenesis (permanent genomic modification). Genetic control also reduces background signal from sources not under scrutiny, increasing SNR (Stosiek et al., 2003), and can permit targeting to a population that is anatomically or functionally related. For example, GINAs allow for specific labeling of direct and indirect pathway neurons in the striatum (Cui et al., 2013) as well as excitatory (Bozza et al., 2004; Ziv et al., 2013) and inhibitory (Kaifosh et al., 2013) neurons which co-localize within the same local neural circuit. Second, GINAs make it possible to record from a large population of cells simultaneously with better spatial resolution than the best electrophysiological techniques (Knöpfel, 2012). Studies exploiting this strength have demonstrated neural network characteristics that were not previously apparent from single cell recordings (Dombeck et al., 2010; Harvey et al., 2012). Finally, GINAs allow for chronic interrogation of the same cells for long periods of time (Mank et al., 2008; Tian et al., 2009; Kuhn et al., 2012; Margolis et al., 2012). As a result, studies which track dynamics of neural circuitry such as learning and memory induced changes to neural ensembles have been made possible (Huber et al., 2012; Petreanu et al., 2012).

Used in conjunction with modern microscopic techniques—most notably two photon (2P) imaging—GINAs are now routinely employed in the interrogation of neural activity under a wide variety of contexts in awake, behaving mammals (Helmchen and Denk, 2005; Bovetti et al., 2014).

## GENETICALLY ENCODED CALCIUM INDICATORS

Calcium dynamics are a proxy to monitor APs in neurons (Tank et al., 1988; Miyawaki et al., 1997) and represent excitable states in astrocytes (Perea and Araque, 2005). In neurons, APs lead to calcium transients in the cytosol through voltage-gated calcium channels (Hille, 2001). This rise is reversed as calcium is buffered, extruded, and pumped back into internal stores in a process which generally lasts on the order of 100 ms (Helmchen et al., 1996; Koester and Sakmann, 2000). Astrocytes, a prevalent type of glial cell in the brain implicated to play important roles in synaptic plasticity, also propagate intracellular signals by means of calcium transients. The source of these calcium events in astrocytes largely overlaps those found in neurons, but due to differential expression levels—particularly a dearth of voltage-dependent calcium channels—and relative cellular location of these sources, calcium events in these cells are longer (generally seconds in duration) and in some cases more highly localized (Ben Achour et al., 2010; Ding, 2013) or show waves of activity in other cases (Hoogland et al., 2009).

Of all GINA classes, GECIs are currently the most widely utilized for *in vivo* imaging in model systems including worm (Kerr et al., 2000; Boulin and Hobert, 2012), zebrafish (Higashijima et al., 2003), fly (Wang et al., 2003), rodent (Hasan et al., 2004; Tian et al., 2009), and recently non-human primate (Heider et al., 2010; Yin et al., 2014). In large part, the success of GECIs has been due to their high SNR (Mank et al., 2008; Chen et al., 2013b) via extensive protein engineering efforts to significantly improve their intrinsic properties, such as brightness (Tian et al., 2009), pH insensitivity (Miyawaki et al., 1999), stable folding (Tallini et al., 2006), photostability (Tian et al., 2009), large dynamic range (Chen et al., 2013b), fast kinetics (Sun et al., 2013) and appropriate expression level (Miyawaki et al., 1999), to match with extrinsic parameters of calcium dynamics in neurons. A summary of GECI design is shown in **Figures 1A–C**.

The first GECIs to gain wide usage were the FRET-based Cameleons which contain M13 and CaM in between a blue/green or cyan/yellow FP pair, conceptually similar to YC-Nano as pictured in **Figure 1B**. Several incrementally improved variants of Cameleon have since been engineered, e.g., the YCs series (for review, see Miyawaki, 2011; Palmer et al., 2011), the computationally redesigned variants D1cpv, D2cpv, D3cpv, and D4cpv to reduce impact on endogenous calcium handling and interaction with endogenous proteins (Palmer et al., 2006), and the high-affinity YC-Nano series (Horikawa et al., 2010). Additionally, a family of GECIs has been developed based on the muscle-specific Ca ^2++^ -binding protein Troponin C: TN-L15, TN-XL (Mank et al., 2006), TN-XXL (Mank et al., 2008), and the recent Twitch sensor (Thestrup et al., 2014). The troponin family in theory has the lowest probability of having interaction with endogenous proteins in cells. In addition, FP pairs have been optimized or replaced as newer, more advantageous FPs became available (Ai et al., 2014).

Meanwhile, a proliferation of single wavelength calcium probes (Camgaroo, Pericam, Case, and GCaMP family) based on enhanced yellow fluorescent protein (EYFP) or circular permutated green fluorescent protein (GFP) have been engineered (Baird et al., 1999; Nagai et al., 2001; Nakai et al., 2001; Souslova et al., 2007). Since then, several papers have been published on the iterative improvement of the GCaMP scaffold, which include GCaMP1.6 (Ohkura et al., 2005), GCaMP2 (Tallini et al., 2006), GCaMP3, and recently developed highly sensitive GCaMPs such as GCaMP-HS (Muto et al., 2011), Fast-GCaMPs (Sun et al., 2013) and GCaMP5 and GCaMP6 series (i.e., GCaMP6s, GCaMPm, and GCaMPf; Chen et al., 2013b). It is noted that a different group uses a distinct numbering system for GCaMP variants which include GCaMP6 (Ohkura et al., 2012), GCaMP7a (Muto et al., 2013), and GCaMP8 (Ohkura et al., 2012). However, these GCaMPs are not incremental improvements upon the GCaMP6 series (Chen et al., 2013b).

With the demonstration that a YC (D3cpv) can readily detect a single AP in mammalian neurons, both *in vitro* and *in vivo* (Wallace et al., 2008), various members of the YC [especially YC3.60 (Nagai et al., 2004)], TN-L [especially TN-XXL (Mank et al., 2008)], and GCaMP [especially GCaMP3 (Tian et al., 2009) and GCaMP5 (Akerboom et al., 2012)] lineages have seen extensive use in *in vivo* preparations. Recent additions to the YC [YC-Nano (Horikawa et al., 2010)], TN-L [Twitch (Thestrup et al., 2014)], and GCaMP families [GCaMP6(s,m,f) (Chen et al., 2013b)] have achieved the long-standing goal of single spike detection in pyramidal neurons on par with or surpassing synthetic dyes in the neurons of living rodents. A summary of performance of GECIs for spike detection in *in vivo* mammalian preparations is presented in **Table 1**.

**Table 1:** Selected examples of modern genetically encoded indicators and their degree of utility in *in vivo* imaging in the mammalian brain.

Genetically encoded indicator lineage/member	Mammalian system *in vivo*	Spontaneous activity detection	Single spike detection	No population or trial averaging	Example reference
***Genetically encoded calcium indicators***					
GCaMP					
GCaMP3	Yes	Yes	No	Yes	Tian et al. (2009)
GCamP5	Yes	Yes	Yes	Yes	Akerboom et al. (2012)
Fast-GCaMPs	No	Yes	Yes	Yes	Sun et al. (2013)
GCaMP6(s,m,f)	Yes	Yes	Yes	Yes	Chen et al. (2013b)
Yellow Cameleon					
YC2.60	Yes	No	Yes	Yes	Yamada et al. (2011)
YC3.60	Yes	No	Yes	Yes	Lütcke et al. (2010)
D3cpV	Yes	Yes	Yes	Yes	Wallace et al. (2008)
YC-Nano	Yes	Yes	Yes	Yes	Horikawa et al. (2010)
TropininC-based					
TN-XXL	Yes	ND	ND	ND	Mank et al. (2008)
Twitch	Yes	Yes	Yes	Yes	Thestrup et al. (2014)
***Reporters of synaptic activity***					
pHluorin-based					
synaptopHluorin	Yes	Yes	ND	No	Bozza et al. (2004)
GluSnFR					
SuperGluSnFR	No	ND	ND	ND	Hires et al. (2008)
iGluSnFR	Yes	Yes	ND	Yes	Marvin et al. (2013)
***Genetically encoded voltage indicators***					
FlaSh					
Flare	Yes	Yes	ND	No	Ahrens et al. (2012)
VSFP					
VSFP2.3	Yes	Yes	No	Yes	Akemann et al. (2010)
VSFP-Butterfly 1.2	Yes	Yes	ND	No	Akemann et al. (2013)
ArcLight					
ArcLight	No	Yes	Yes	Yes	Cao et al. (2013)
Rhodopsin-based					
MacQ	Yes	Yes	Yes*	Yes	Gong et al. (2014)
ASAP					
ASAP1	No	ND	ND	ND	St-Pierre et al. (2014)

*Detected long-lasting Ca^2+^ spikes only. Shorter duration Na^+^ spikes were not resolved.

*ND, not determined.*

Another exciting advance in GECI development in the past few years has been the expansion of new color-palette variants, such as the red-shifted variant R-GECO (Zhao et al., 2011) based on mApple and RCaMP based on mRuby (Akerboom et al., 2013). Extending the color-spectrum has greatly increased the potential of GECIs in multiplex imaging. Multi-color imaging allows simultaneous assay of diverse cell types. For example, different color probes will aid in the elucidation of the interplay between neurons and astrocytes in shaping the neural circuitry. Red-shifted indicators will additionally reduce tissue scattering (for both excitation and emission), phototoxicity, and background fluorescence, facilitating deep imaging. Finally, color-shifted indicators will seamlessly integrate into imaging experiments with other types of indicators and optogenetic tools (for review, see Akerboom et al., 2013).

For the past years, GECIs have driven the expansion of knowledge about the dynamics of neural circuitry gained through *in vivo* imaging of the intact brain (For details, see Recent Findings through GINA Technologies). GECI variants continue to open up new applications in neuroscience. Transgenic expression of inverse pericam and camgaroo-2 allowed the first detection of calcium signals evoked via sensory manipulation in a single trial in mouse (Hasan et al., 2004). D3cpv produced the first recordings of single spikes from mouse cortical cells *in vivo* (Wallace et al., 2008). Several GINAs have been deployed under a glial fibrillary acidic protein (GFAP) promoter in mice to allow readout of astrocytic activity in acute slice (Haustein et al., 2014) and in the intact, anesthetized animal (Atkin et al., 2009; Hoogland et al., 2009). Members of the TN-L (TN-XXL; Heider et al., 2010) and GCaMP (GCaMP5; Yin et al., 2014) lineages have recently been deployed for imaging studies in non-human primates. Future work within this model organism will produce results more pertinent to the human nervous system. GCaMP6 was used to report calcium transients from dendritic spines of excitatory and the dendrites of inhibitory interneurons located in primary visual cortex (Chen et al., 2013b). All of these milestones continue to demonstrate that GECIs have been a useful tool for breaking down barriers to advance our understanding of the dynamics of neural circuitry.

Despite the fact that calcium transients are an indirect measure of systems level circuit function, their superior SNR values relative to other GINA classes have assured their continued dominance in this arena for the near future. Though the advance of monitoring circuitry function depends on the improvement of genetically encoded voltage indicators (GEVIs) and other sensors probing all kinds of neural activity—for example transient changes to neuromodulator concentration in the extracellular space—GECIs will continue to find utility in the study of electrically silent cells which interact with brain circuitry, such as astrocytes.

## SENSORS REPORTING SYNAPTIC ACTIVITY

Rapid information flow in the brain is mediated by anatomical connections at synapses between cells. During the course of an AP, membrane depolarization invades the presynaptic terminal. As noted above, this results in intracellular calcium transients, which in turn drive the fusion of neurotransmitter containing vesicles with the presynaptic cell membrane. This fusion event results in an immediate increase in the vesicular pH, which is maintained at an acidic level, as the lumenal volume is expelled into the extracellular space. The contents of the vesicle are also released into the synaptic cleft, leading to a local increase in the concentration of neurotransmitter (Hille, 2001).

To better access synaptic transmission with optical tools, a variety of GINA classes have been developed. The first of these, synaptopHluorin, is derived from the fusion of a pH sensitive, ecliptic GFP variant at the c-terminus of the vesicular protein, synaptobrevin [also known as vesicular associated membrane protein-2 (VAMP2)], which localizes the FP to the vesicular lumen. The fluorescence of the ecliptic pHluorin is quenched by the acidic environment of synaptic vesicles (pH 5.6). During neurotransmitter release, vesicles fuse with the plasma membrane, exposing the lumen to the neutral pH of the extracellular environment (pH 7.4), causing a dramatic increase in fluorescence intensity. The fluorescence intensity is then quenched once again after reconstitution and reacidification of the vesicle interior (Miesenbock et al., 1998; **Figure 1D**). SynaptopHluorin was one of the first GINAs to be employed for *in vivo* imaging in the mammalian system (Bozza et al., 2004).

Later modifications to these probes included fusions to different proteins which localize with higher specificity to synaptic vesicles such as synaptophysin (Granseth et al., 2006) and the vesicular glutamate transporter (VGLUT-1; Balaji and Ryan, 2007). Red-shifted variants of synaptopHluorin have also been developed for multiplex imaging. These were constructed by exchanging the GFP based pHluorin with relatively red shifted FPs and include VGLUT1-mOrange2 (Li et al., 2011) and sypHTomato (Li and Tsien, 2012). Each of these probes was used concurrently with a GCaMP indicator to simultaneously probe presynaptic calcium signaling and presynaptic release at the same synapses or in distinct pre- and postsynaptic elements. These studies serve as early examples of the future possibilities presented by multiplex imaging.

Genetically encoded indicators of neural activity for probing excitatory neurotransmitter glutamate release and spillover have also been developed. Fluorescent indicator protein for glutamate (FLIPE; Okumoto et al., 2005) and the SuperGluSnFR (Tsien, 2005; Hires et al., 2008) are FRET-based probes which employ the linear fusion of periplasmic glutamate binding protein of *Escherichia coli*, GltI, (also known as ybeJ) with enhanced cyan fluorescent protein (ECFP) and a yellow FP, Citrine or Venus (**Figures 1E,F**). These reporters provide a sensitive optical readout of glutamate concentration *in vitro* by FRET-dependent changes in the CFP/YFP emission ratio. An improved version of GluSnFR, SuperGluSnFR has since been developed. Through linker optimization (Hires et al., 2008), SuperGluSNFR permits efficient optical measurements of the time course of synaptic glutamate release, spillover, and reuptake in cultured hippocampal neurons with centisecond temporal and dendritic spine-sized spatial resolution. Most recently, a single-FP based glutamate sensor, iGluSnFR, was developed. iGluSnFR is based on the fusion of Gltl with circularly permuted enhanced green fluorescent protein (cpEGFP; **Figure 1E**). Due to the superior SNR of this probe, it allows the resolution of glutamate transients at the apical tuft dendrites of layer V neurons in the motor cortex of awake, behaving mice (Marvin et al., 2013).

The development of sensors probing synaptic activity greatly expanded the kinds of neural activity that can be accessed by optical tools. The output activity of a neuron is sometimes thought of as being the voltage transients propagated to synaptic termini during an AP. Calcium transients are currently the most experimentally accessible marker of this phenomenon. However, under some conditions, the relationship between these signals and synaptic release is non-linear (see, e.g., Singer and Diamond, 2006). In this respect, reporters of synaptic activity will sometimes be a better choice of sensor for applications in which vesicle/transmitter release is the primary concern. Furthermore, neurotransmitter sensors allow detection of the presence of extra-synaptic neurotransmitter, which can have important functional consequences when interacting with neuronal receptors expressed distal to synapses (Marvin et al., 2013) and on astrocytes (Haustein et al., 2014).

## GENETICALLY ENCODED VOLTAGE INDICATORS

Membrane potential undergoes a rapid change during the course of an AP as well as subthreshold oscillations. These changes can be succeeded by consequent changes to intracellular calcium and evoked synaptic signaling events (Hille, 2001). As such, voltage is considered the primary signal of interest and the most direct way to monitor neural activity. Despite this fact, imaging voltage is inherently difficult, largely due to the nature of the voltage signal itself. The short duration of the voltage change during APs (1–5 ms) demands faster kinetics and higher sensitivity of fluorescent sensors to yield sufficient photon budget for imaging (Peterka et al., 2011; Looger and Griesbeck, 2012). Additionally, GEVIs are necessarily membrane bound or associated, which reduces imaging volume relative to cytosolic GINAs (Peterka et al., 2011). However, recent advances in the field of protein engineering hold great promise for the development of GEVIs with superior SNR.

As demonstrated in **Figures 1G,I,J**, most GEVIs function as reporter of membrane voltage by tethering one or two FPs to a voltage sensitive protein derived from natural sources. The first genetically encoded voltage indicators—such as FlaSh (Siegel and Isacoff, 1997), sodium channel protein-based activity reporting construct (SPARC; Ataka and Pieribone, 2002), and voltage sensitive fluorescent protein 1 (VSFP 1; Sakai et al., 2001)—were based on intact voltage-gated potassium channels or their “voltage-sensing” domains. However, these probes exhibit low SNR, slow kinetics and localize poorly to the membrane of mammalian cells (Baker et al., 2007).

The next generation of GEVIs was based on the voltage sensitive domain of a phospatase (VSP) derived from the sea squirt, *Ciona intestinalis* (Ci-VSP; Murata et al., 2005; Dimitrov et al., 2007; Akemann et al., 2010; Jin et al., 2012; **Figures 1G,H**). These probes include both FRET-based (Akemann et al., 2010) and single FP-based family (Lundby et al., 2008; Perron et al., 2009), and boasted much improved brightness, kinetics and improved membrane targeting relative to the earlier generation. One of the most advanced probes of this class, VSFP2.3, permitted *in vivo* recording of neural activity in mouse somatosensory cortex, despite poor cellular resolution. Recently, an improved variant, VSFP butterfly 1.2 (**Figure 1G**), showed increased SNR and allowed two-photon imaging of membrane changes produced by layer2/3 neurons of mouse barrel cortex, which represents the first reported use of at depth cellular resolution imaging by a GEVI. However, extensive trial averaging was required in order to resolve the signal (Akemann et al., 2013).

In parallel, protein engineering efforts have also led to single-FP based GEVIs. For example, the ArcLight (**Figure 1H**) fluorescence voltage sensors consist of the fusion of ecliptic pHluorin GFP to Ci-VSP and show significantly improved sensitivity in response to APs compared to other Ci-VSP based probes (Cao et al., 2013). More recently, Accelerated Sensor of Action Potentials 1 (ASAP1) has been developed in which circularly permuted green fluorescent protein (cpGFP) is inserted in an extracellular loop of a voltage-sensing domain of a VSP derived from a chicken (*Gallus gallus*). ASAP-1 showed faster kinetics compatible with the typical 2-ms duration of APs and is capable of probing high-frequency AP trains (St-Pierre et al., 2014), thus permitting detection of subthreshold potential and hyperpolarization waveforms in cultured hippocampal neurons. However, voltage imaging using both ArcLight and ASAP-1 in intact rodent brains remains to be demonstrated.

In addition, another recent class of GEVI including PROPS (Kralj et al., 2011) and VIP1 (Kralj et al., 2012), has been developed based on voltage-induced fluorescence modulation of the retinal cofactor of bacterial and archaeal rhodopsins. In these probes, the rhodopsin itself acts as the sensor domain while the retinal cofactor serves as the fluorescence reporter *via* direct fluorescence (Kralj et al., 2011, 2012; Gong et al., 2013) or acting as FRET acceptor (illustrated in **Figure 1H**; Gong et al., 2014). Relative to the Ci-VSP based sensors, this class of sensor is extremely fast with submillisecond kinetics, sufficient to resolve single AP or subthreshold membrane voltage fluctuations in cultured mammalian neurons. However, because these probes rely on a retinal cofactor as the sole fluorescence source, they exhibit extremely low brightness. The most recent FRET-opsin sensors offer fast kinetics (rise time ∼5 ms) as well as higher brightness, which has allowed for reporting long-lasting complex spikes in the dendritic arbors of Purkinje cells in the cerebellum of a living mouse. These spikes exhibit slower dynamics than typical Na^+^ spikes (∼5–10 ms), but nevertheless were apparently detected at sub-cellular resolution with single-trial precision (Gong et al., 2014), a first for this GINA class.

Though current state-of-the art GEVIs have been deployed for *in vivo* imaging, their broad use has remained limited in rodent brain as shown in **Table 1**. As incremental improvements are made to existing and future probes, GEVIs may finally facilitate systems-level, cellular-resolution voltage imaging in living behaving mammals.

Blogs discussing all GINA classes can be found at open websites such as Andrew Hires’ Brain Windows^1^ and Guillame Dugué’s OpenOptogenetics^2^.

## CASE STUDY: FROM GCaMP3 TO GCaMP6

**FIGURE 2:**
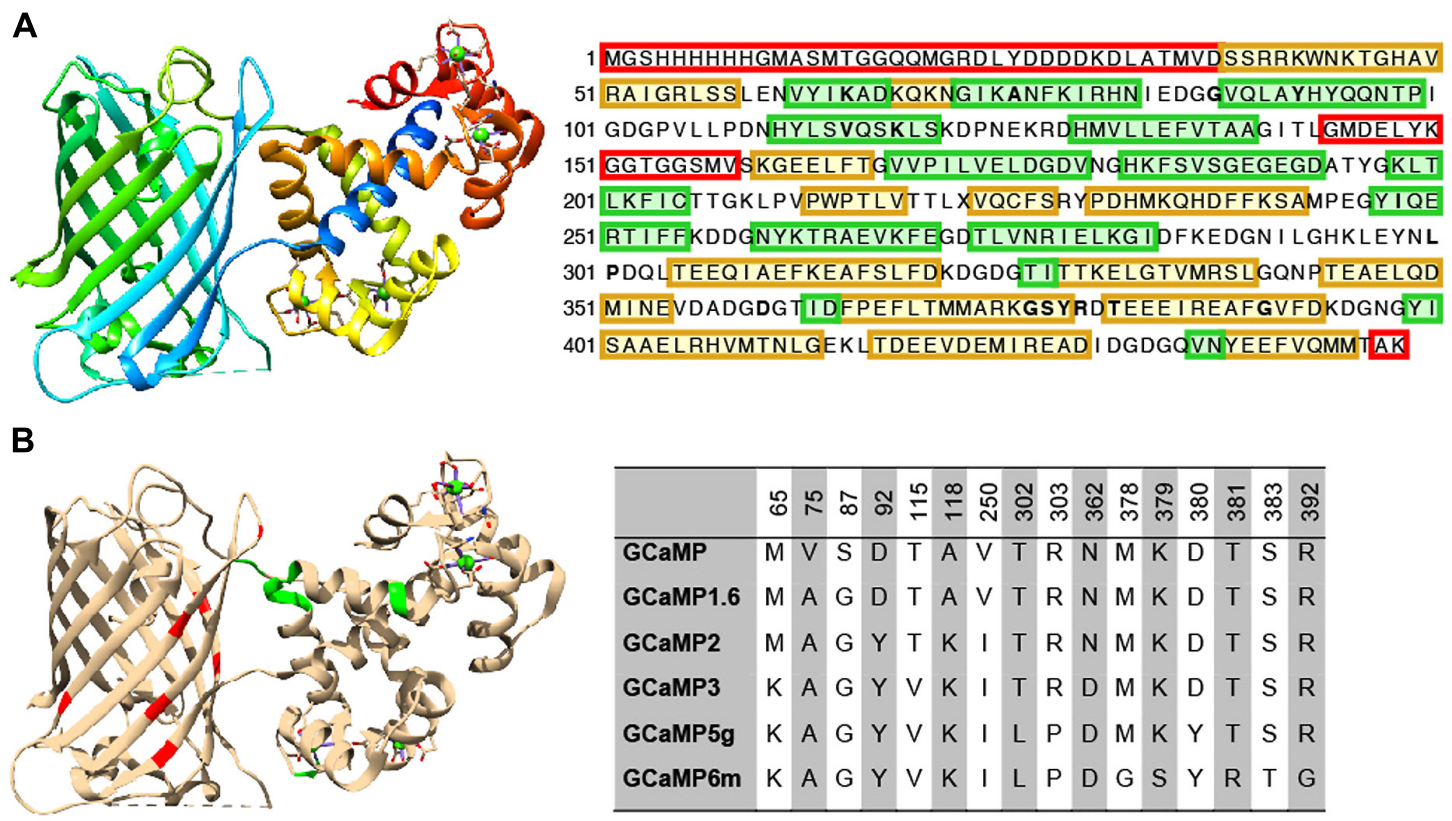
**Structure and beneficial mutations of GCaMPs. (A)** Crystal structure (Ding et al., 2014) and primary sequence of GCaMP6m. Distinct regions of the crystal structure are indicated by color. The cooler the color, the closer the sequence is to N-terminus. The primary sequence is annotated such that yellow shading corresponds to ß-strands, green shading corresponds to α-helices, and residues in red are those that do not crystallize (i.e., pRSET domain and linkers). **(B)** Beneficial mutations of GCaMPs. Crystal structure of GCaMP6m is annotated with iteratively evolved residues. Red residues are from the cpGFP and green ones are from calmodulin. Note that these residues are typically at the interface between the two domains. Residue matrix shows the evolution of GCaMP lineage with incrementally improved performance. Individual beneficial residues are highlighted in the primary sequence of GCaMP6m in **(A)**.

Extensive protein engineering efforts have improved the properties of GINAs, especially the GCaMP family, to the point that they now rival synthetic alternatives such as Oregon Green Bapta-1 in terms of kinetics and sensitivity. Here we review established sensor design and optimization pipelines (rational design combined with directed evolution) that have proven to be effective to improve the intrinsic properties of GCaMPs, leading to a family of high performing sensors, GCaMP3, GCaMP5, and GCaMP6(s,m,f; **Figures 2A,B**).

Based on GCaMP3 scaffold, an array of GCaMP5 variants was generated by combining improvements from site-directed mutagenesis at cpEGFP/CaM interface and targeted library screening at the two linkers between cpEGFP and M13/CaM (Akerboom et al., 2012). Twelve GCaMP5s were systematically characterized in cultured neurons, astrocytes, and in *Caenorhabditis elegans*, drosophila, zebrafish, and mouse under various conditions. SNR was improved by at least twofold to threefold; all GCaMP5s showed improved dynamic range compared with GCaMP3. Users can choose from different GCaMP5 variants for different applications. Amongst these, GCaMP5A, GCaMP5G, and GCaMP5K exhibit the highest levels of general utility.

Based on the GCaMP5 scaffold, computational design has guided further targeting of 16 amino acid positions on the interface between cpEGFP and CaM at 18 positions including the M13-CaM interface (Chen et al., 2013b). Four hundred and forty seven GCaMP variants in were screened in dissociated neuronal culture resulting in three ultrasensitive GCaMP6 sensors with a variety of kinetics [i.e., GCaMP6s (slow), GCaMP6m (medium; **Figure 2A**), and GCaMP6f (fast)]. Compared to GCaMP5G, these sensors have 1.1- to 1.6-fold increase of dynamic range and threefold higher Ca^2+^ binding affinity. In addition, GCaMP6f shows the fastest kinetics among the entire GCaMP family due to a mutation at the M13-CaM interface (A317E).

Besides protein engineering efforts, the success of GCaMP6 development also depended on the establishment of a highly efficient high-throughput screening platform (Wardill et al., 2013). This system directly employed screening in dissociated rat cortical/hippocampal neuronal culture, bypassing the screening steps in bacterial and mammalian cells (Tian et al., 2009; Zhao et al., 2011). Specifically, lentivirus encoding GCaMP variants driven by human *synapsin-1* promoter were produced to achieve dense and neuron-specific labeling in cultured neurons. To assist automated processing of imaging acquisition and analysis, internal ribosome entrance site (IRES) were introduced downstream of GCaMP to drive the expression of a second FP, nuclear targeted mCherry. The process of screening has been automated from probe development to imaging analysis (Wardill et al., 2013). This screening system can be easily adapted to aid in the optimization of other members of the GINA family.

As new members of the GCaMP family and other GINA classes are developed, their creators will often make them available for the larger research community. Frequently, genetic material encoding the probes can be acquired through Addgene^3^, a non-profit plasmid repository. For ready-made application, GINAs packaged into viral vectors can be obtained through viral core facilities of institutions such as the University of Pennsylvania^4^ and the University of North Carolina at Chapel Hill^5^. Finally, animals transgenic for several GINAs have been produced and are available for purchase through services such as those provided by the Jackson Laboratory^6^ and the Zebrafish International Resource Center^7^.

## *IN VIVO* IMAGING WITH GINAs IN LIVING, BEHAVING RODENT BRAIN

Though some classes of GINAs have been optimized to the point that they are compatible for *in vivo* imaging, the noise level *in vivo* greatly challenges the SNR of the sensors, especially in the mammalian brain. To make imaging experiments meaningful and successful, consideration must be taken for proper selection of imaging configuration, expression systems, surgical procedure, imaging depth, and analytical techniques. To provide guidance for the end-users for choosing most suitable experimental settings, here we review a few important intrinsic and extrinsic parameters that are essential to maximize SNR and to achieve best imaging outcomes.

### GINA TRANSDUCTION METHODS

The expression level of a GINA influences its SNR in cells and organisms in a complex manner. Low expression level will preclude sensor visualization and decrease SNR. It also demands imaging with higher laser power and longer exposure time, which may lead to phototoxicity, reduced imaging speed, and increased photobleaching. On the other hand, high expression level increases photon budget, but also increases substrate buffering (e.g., calcium by GECIs) and may perturb cell signaling pathways leading to cytotoxicity and cytomorbidity (see, e.g., Kuhn et al., 2012). Variation of expression level from cell to cell also impacts sensor performance, potentially confounding quantitative imaging analysis. To balance all these effects, it is essential to explore multiple promoters and regulatory sequences combined with the most suitable gene delivery methods to maximize the SNR of the sensor. Several methods of gene delivery have been optimized for the expression and subsequent recording of GINAs in the intact mammalian brain. These include introduction via stereotaxic viral injection, *in utero* electroporation, and stable transgenesis.

In the case of viral gene delivery, virus-encoding GINAs are injected directly into the brain region of interest (Wallace et al., 2008). Typically, recombinant adeno-associated virus (rAAV) has been broadly used due to its ability to diffuse easily away from the site of injection and relative simplicity (Grieger and Samulski, 2005). But others, such as adenoviral vector (e.g., Mohamed et al., 2013) and lentiviral vectors (e.g., Akerboom et al., 2012), are also in use (Packer et al., 2013). Combined with tissue specific promoters, such as synapsin-1, viral injection permits labeling of genetically defined local populations of neurons. The efficiency of probe expression can vary as a function of cell type, promoters, brain regions, and AAV serotype (Cearley and Wolfe, 2006; Wallace et al., 2008; Kuhn et al., 2012; Aschauer et al., 2013). For example, rAAV2 drives differential probe expression in cerebellar interneurons (Kuhn et al., 2012). However, when combined with specific promoter system, rAAV can be targeted to Purkinje cells in cerebellum cortex (Kuhn et al., 2012). In addition, rAAV9 is well suited for the transduction of cortical neurons (Cearley and Wolfe, 2006), while rAAV8 is particularly efficient in labeling astrocytes (Aschauer et al., 2013). Hybrid viruses have been developed which increase potential combinations of transduction efficiency and tropism. For example, AAV2/1 encoding GINAs have been widely used because they display a broad tropism within the central nervous system combined with high transduction efficiencies (Burger et al., 2004). Viral labeling results in long-term, relatively stable probe expression over months, during which time imaging experiments can be performed repeatedly (Mank et al., 2008; Tian et al., 2009; Kuhn et al., 2012; Margolis et al., 2012). The popularity of this method of gene transfer is due in large part to its relatively easy adoption with flexibility in choosing tool payload and injection locations.

*In utero* electroporation is a process in which plasmid is injected, usually to the ventricles of an animal during the prenatal period of its life. The plasmid is driven into cells at the ventricular surface via application of electrical current across the target structure. Due to the temporal pattern of laminar development and spatial pattern of cell lineages present in the cortex, the time and location at which the procedure is performed can restrict probe expression to specific layers (Tabata and Nakajima, 2001) or cell populations (Borrell et al., 2005) within the cortex. However, the expression level of GINAs driven by this method has been reported to cause cytotoxicity as expression begins during a developmentally sensitive period (Tian et al., 2009). Furthermore, the variation of expression level in cells appears more pronounced than that using viral transduction. Still, studies that are based around developmental questions of circuit formation can benefit from the use of this method.

Transgenesis refers to the permanent modification of the genome of an organism by the use of, for example, bacterial artificial chromosomes (Van Keuren et al., 2009), zinc finger nucleases (Urnov et al., 2010), transcription activator-like effector nucleases (Joung and Sander, 2013), or clustered regularly interspaced short palindromic repeats (CRISPR) associated genes (Mali et al., 2013). Transgenic rodent lines that stably express GINAs through multiple generations have been a standard tool in neuroscience. Transgenic lines have been engineered expressing GINAs such as inverse pericam and camgaroo-2 (Hasan et al., 2004), synaptopHluorin (Bozza et al., 2004), GCaMP2 (Díez-García et al., 2005), YC3.60 (Atkin et al., 2009), the hybrid voltage sensor (hVOS; Wang et al., 2012), and GCaMP3 under control of the Thy1 promoter (Zariwala et al., 2012). Gene transfer through transgenesis results in the stable and ubiquitous expression of the genetic construct in target cells throughout the organism, although expression levels tend to vary across lines and tissue regions (Zeng and Madisen, 2012). Studies that require dense sampling from a genetically defined population over the life course of an organism benefit from the use of transgenic organisms.

Targeting GINAs with adequate cell type specificity can be achieved through recombinase systems such as the Cre/loxP and FLP/FRT, especially by pairing recombinase-dependent viruses with recombinase-expressing animal lines. Selective expression of GINAs in subsets of anatomically or functionally related populations is possible by placing the GINA construct into the double-floxed inverted orientation (DIO) configuration (Atasoy et al., 2008) or using recently developed INTRSECT (Intronic recombinase sites enabling combinatorial targeting) strategy in which expression is contingent upon the presence of Boolean subsets of transcription factors (Fenno et al., 2014).

Pharmacological control can be exerted to achieve temporal control of GINA expression. For example, the Tet-On(Off) system causes expression(repression) of GINA expression in the presence of tetracycline or one of its analog (Gossen and Bujard, 1992; Hasan et al., 2004). Such temporal control can be important to prevent perturbation of early developmental processes or animal behavior, which may confound imaging results (Tian et al., 2009).

### OPTICAL INSTRUMENTATION

One of the most vital components for successful *in vivo* fluorescence imaging in the mammalian brain is the microscopic system. Brain tissue is highly scattering to light and contains fluorophores, for example flavoproteins, with spectra which can overlap to a high degree with those of current GINAs. At the same time, the neural activity which is tracked by GINAs unfolds on the order of 1s to 100s of milliseconds, limiting the time-window for photon capture. Fluorescence microscopy including systems based on one photon (1P) or multiple photons have been developed and optimized to facilitate imaging experiments.

In one photon fluorescence microscopy, a fluorophore is initially bombarded with photons from a relatively high energy excitation light source. After absorption, some of the energy of the photon is lost through non-radiative processes. The fluorophore then emits lower energy photons which can be detected. Visible light employed in 1P microscopy scatters more easily in tissue because mean path length of a photon increases shorter wavelength. This effect limits the depth at which 1P systems are capable of imaging unaided to ∼100 μm. However, charge-coupled device and complementary metal oxide semiconductor detectors which can be used in conjunction with this imaging modality are capable of extremely fast (>kHz) full field image acquisition rates. They are also typically relatively inexpensive when compared with comparable multi-photon alternatives (Helmchen and Denk, 2005).

In addition, 1P imaging light paths can be channeled through endoscopes (Flusberg et al., 2008; Barretto et al., 2011) as well as single and multimode fiber optic cable (Ferezou et al., 2006; Flusberg et al., 2008; Lütcke et al., 2010; Gunaydin et al., 2014) to image cells at depth within brain tissue. These systems can be deployed to image the brains of head-immobilized animals (Akemann et al., 2010) or miniaturized and mounted to a freely moving animal (Ghosh et al., 2011). As a note of caution, the introduction of an endoscope to brain tissue is highly invasive, while fiber-optic techniques generally result in data nearly devoid of fine-scale spatial information (although see, e.g., Ferezou et al., 2006), in which a braided fiber optic cable allowed the acquisition of coarse-grained spatial data).

Multi-photon—and in particular 2P—microscopy has been the workhorse for *in vivo* imaging in the field of systems neuroscience (Denk et al., 1990). This is due primarily to two attractive, intrinsic features of these systems to reduce out of focus emission, light scattering, and phototoxicity. First, in multi-photon microscopy photon absorption varies non-linearly with excitation photon density. For example, this relationship is quadratic in the case of 2P microscopy. As a result, only fluorophores within the focal volume of the excitation light cone are activated. Second, the energy of multiple photons must combine to excite the fluorophore, meaning that lower energy (and thus, less scattering) excitation photons may be used (Denk et al., 1990).

Traditional 2P systems are limited in acquisition speed by the galvanometer mirrors, which give rise to low frame rate (up to 15 Hz at 512 × 512 frame size), but full flexibility of arbitrary scan geometries within a two-dimensional plane. Such geometric patterns can be extended into three dimensions by controlling axial motion with a piezoelectric focusing element, allowing fast, single line scans within this space (Göbel et al., 2007). Alternatively, resonant laser scan mirrors enable very fast scanning of high resolution full field frames (512 × 512 pixels) at 30 fps or higher with 8 kHz scanners (Denk et al., 1990; Helmchen and Denk, 2005; Kerr and Denk, 2008). However, arbitrary scan patterns cannot be accommodated with a resonant mirror.

Imaging depths of ∼500 μm using GINAs have been achieved routinely in 2P imaging while maintaining excellent spatial resolution in intact mammalian preparations (Helmchen and Denk, 2005). This depth has been increased through the use of adaptive optics, regenerative amplifiers (Theer et al., 2003; Mittmann et al., 2011) and three photon microscopy (Ouzounov et al., 2014) to ∼1000 μm, though the imaging depth can be increased up to ∼1600 μm using small molecule dyes (Kobat et al., 2011). Greater depths can be achieved by using a fiber optic light path in conjunction with a piezoelectric driven actuator for raster scanning through the fiber (Helmchen et al., 2001; Flusberg et al., 2005; Sawinski et al., 2009). The relatively slow speed of image acquisition can be addressed through random access scanning of small regions of the volume of a cell or field of cells using acousto-optic devices to control the excitation scan path (Grewe et al., 2010; Katona et al., 2012; Fernández-Alfonso et al., 2014). Alternatively, multiple excitation beams can be multiplexed for near- simultaneous imaging of different spatial patterns (Cheng et al., 2011).

Widefield illumination with multi-photon techniques can be achieved through the use of spatial light patterning techniques such as spatial light modulation (Nikolenko et al., 2008) and the generalized phase contrast method (Palima and Gluckstad, 2008) as well temporal focusing (Oron and Silberberg, 2005; Papagiakoumou et al., 2010). When employed in conjunction, such techniques allow simultaneous excitation of large, 3D volumes. The resultant emission patterns can then be interrogated through a typical widefield microscopic detection apparatus (Papagiakoumou et al., 2010).

As imaging technologies such as these become more developed and widespread, GINAs will become ever more useful. Microscopes which are mountable to an unrestrained animal will allow recordings from animals in more ethologically relevant states. Technologies that produce faster frame rates in 2P imaging will allow denser sampling of neurons within a single recording epoch with current GECIs. These technologies will further be indispensable for constructing accurate recordings of future GEVIs expressed in mammalian systems.

### ACQUISITION OF MICROSCOPIC IMAGES

Due to its propensity to scatter light, the skull presents a significant barrier to imaging an intact brain. However, it also serves to dampen pulsations arising from the movement of blood and cerebrospinal fluid through cerebral tissue. As a result, simply removing the cranium above the imaged region is not a common practice in modern GINA imaging. Typically, the skull’s impact on image quality is reduced by either thinning the skull above the imaged region(s) (Grutzendler et al., 2002) or by inserting a glass cover slip above a craniotomy (Holtmaat et al., 2009). Both methods stabilize cellular position within brain tissue while allowing high-quality optical access.

Motion artifacts can further complicate imaging acquisition in living animals. Multiple approaches have been devised to reduce motion artifacts, including mechanical changes to the imaged tissue, animal restraint, and online and *post hoc* computational processing for maintaining image stability.

For 2P imaging, head fixation techniques to maintain the animal’s position have been used in monkeys (Wurtz, 1968), flies (Dahmen, 1980), and rodents (Komisaruk, 1970). Head and body restraint allow stable imaging while both stimulus and behavioral response parameters can be tightly controlled. This, in turn, eases the task of quantifying neural activity with respect to these variables (Akemann et al., 2010; Andermann et al., 2010; Huber et al., 2012; Petreanu et al., 2012). Head restraint can be used in conjunction with a spherical (Dombeck et al., 2007) or linear (Marvin et al., 2013) treadmill, which allows the addition of assays which require locomotion on the part of the animal. Animals restrained in this manner can then be presented with passive stimuli (Niell and Stryker, 2010) or virtual sensory environments in which they can exert control over the stimulus via movement of the treadmill (Dombeck et al., 2010).

The microscope objective itself can also be stabilized relative to the imaged specimen via closed-loop designs whereby movement of the animal results in registered movement of the relevant optics. These systems function by detecting and correcting for motion in the axial direction based on general movement of tissue (e.g., Laffray et al., 2011) or by modeling and avoiding (Paukert and Bergles, 2012) or counteracting (Chen et al., 2013a) movement due to specific motion artifacts relevant to the experimental settings.

In addition, a number of algorithms have been developed for motion correction. These algorithms function by appropriately reshaping images in a time series such that consecutive frames are aligned. Algorithms developed for this purpose are based on Hidden Markov Models (Dombeck et al., 2010), cross-correlation based registration (Guizar-Sicairos et al., 2008), or gradient-descent based minimization of image differences (Greenberg and Kerr, 2009). Besides computational approaches, FRET sensors or single FP sensors expressed along with a reference FP can be used to cancel the effects of motion artifact.

### IMAGE PROCESSING

Image processing is a necessary step, as signals must be assigned to specific cells or subcellular compartments and described according to their content. A large array of approaches for analyzing and visualizing GINA data has been developed. One of the first questions addressed using such methods was image segmentation (i.e., what parts of the imaged field contain responsive elements) and spike or event detection (i.e., when does the fluorescence contained within these elements change significantly from baseline). Such approaches reduce variability and bias introduced to analysis by methods such as hand drawing of regions of interest (ROIs). Early algorithms developed for spike detection were based on simple thresholding of the ΔF/F signal [i.e., (*F* - *F*_0_)/*F*_0_ where F is the moment by moment spatially averaged fluorescence data and F_0_ is typically defined as a time-averaged, background subtracted baseline fluorescence value] where event detection was triggered by this signal passing above the threshold (e.g., Mao et al., 2001). Later efforts have employed linear deconvolution (Yaksi and Friedrich, 2006; Holekamp, 2008; Vogelstein et al., 2010) or subtraction (Seelig et al., 2010) to detect occurrence of spiking events. These algorithms perform well in systems in which probe response is linear to the input signal. Other methods have used pixel-wise correlation in the fluorescence signal (Ozden et al., 2008), principle components analysis followed by independent components analysis (Mukamel et al., 2009), Monte Carlo spike detection (Vogelstein et al., 2009), and supervised learning algorithms (Valmianski et al., 2010).

As the size of imaging datasets grows ever larger, it becomes increasingly critical to extract salient features within and across populations of cells expressing GINAs, especially correlating the activity of cells to the behavioral output of an animal. Such approaches can correlate the activity of single cells to behavioral data by simple coincidence (Seelig et al., 2010), regression (Miri et al., 2011), or machine-learning based classification schemes (Huber et al., 2012; Petreanu et al., 2012). Additionally, dynamics of entire cell populations can be visualized in low dimensional space by methods based on factor analysis of the dataset (Harvey et al., 2012). Recently, many of the most successful approaches examined above have been integrated into a suite of open source tools optimized for the analysis of large datasets termed “THUNDER” (Freeman et al., 2014) which can be accessed at http://thefreemanlab.com/thunder/. Taken together, the computational approaches for imaging analysis and visualization represent an indispensable set of tools for interpreting the vast quantities of data acquired in the course of GINA-enabled experiments.

## RECENT FINDINGS THROUGH GINA TECHNOLOGIES

Genetically encoded indicators of neural activity attributes—particularly genetic specificity and long-term expression—make them excellent tools for circuit interrogation at multiple spatial and temporal scales. They have thus been used in recent years to make fundamental contributions to our understanding of brain function. In addition, several GINA classes have made important strides toward becoming standard tools for various applications in the study of mammalian neural circuitry *in vivo*.

### TOPOGRAPHICAL REPRESENTATIONS

Topographical maps are systematic variations in the spatial layout of the response properties of a brain region which reflects the organization of a stimulus space (Hubel and Wiesel, 1959; Felleman and Van Essen, 1991). While electrophysiological techniques have generally been used to develop mesoscopic-scale [order of 100 μm (Knöpfel, 2012)] mappings of brain regions, the application of GINAs in topographic mapping provides single cell spatial resolution. In addition, GINAs make it straightforward to rapidly transition between spatial scales, acquiring data from the cellular or population level or anywhere in between by simply changing image magnification.

The activity of genetically specified elements of a neural circuit can be recorded using GINAs. Topographical mapping of odorant to region of the rodent olfactory bulb has been described using GINAs transgenic animals (e.g., Hasan et al., 2004) or by intrinsic optical signals. Indeed, both techniques report a systematic olfactory map of homologous aliphatic compounds of increasing length (Johnson et al., 1999; Meister and Bonhoeffer, 2001; Fletcher et al., 2009). Only imaging studies with GINA, however, were able to leveraged the genetic specificity of GINAs to discern that the aliphatic odorant map is formed *de novo* within the postsynaptic circuitry of the olfactory bulb (Bozza et al., 2004; Fletcher et al., 2009).

One of the most powerful aspects of GINAs in studying topographical maps is the ability to allow for imaging cellular activity at spatial scales ranging from subcellular to millimeter, providing researcher to inspect the proverbial forest, individual trees, or both, all while maintaining awareness of current location. For example, a conspicuous lack of correlation between CA1 place cell location and the space they encoded has been demonstrated through cellular resolution imaging of virally driven GCaMP3 (Dombeck et al., 2010). In another recent study, optical recording auditory cortex at different spatial scales has resolved coarse and then cellular scale cortical activity in response to different tones. These results support the existence of a tonotopic axis along primary auditory cortex whose strength had been called into questions by previous studies (Issa et al., 2014).

### FUNCTIONAL ORGANIZATION OF NEURAL CIRCUITRY

Local circuits within different cortical regions receive inputs from multiple distant brain regions. These inputs impact ongoing computations within, and thus the output from, local circuitry. In terms of input, a critical question is: what information is conveyed from distal brain regions to the local area? Several recent studies employed GECIs to record calcium transients in distal boutons of, for example cortico-cortical (Petreanu et al., 2012) and medial septal nucleus-hippocampal axons (Kaifosh et al., 2013) to address this question. Such studies have allowed unprecedented access to communication from one brain region to another with both cellular and genetic resolution.

Genetically encoded calcium indicators have also been used in addressing whether response properties of local cells within a region are a function of inputs received from hierarchically lower brain regions, or if those properties arise from local circuit interactions. For example, Glickfeld et al. (2013) demonstrated that the response properties of boutons projected from primary visual cortex in mouse to higher visual areas matched the response properties of cells within each region. This finding stands in contrast to the results from Fletcher et al. (2009) who demonstrated a *de novo* map of olfactory space that arises through local circuit interaction. In each case, GINAs were useful in disambiguating the degree to which local circuit computations result in new response properties.

Taken together, these studies point to the ways in which GINAs aid decoding of communication between brain regions, especially, revealing what message is sent between regions and how that message influences processing within the target structure. As probes of multiple colors become available for *in vivo* use, it will become possible to address such questions within a single experiment.

### STABILITY AND PLASTICITY OF NEURAL CIRCUITRY

Neural circuitry is constantly reorganized structurally and functionally in response to experience and other influences on sensory input. Much effort has been dedicated in recent decades to determining under what conditions neural responses to a stimulus remain stable over time (Gerrow and Triller, 2010).

Genetically encoded indicators of neural activity are particularly well suited to the study of how neural responses to stimuli change over time. Because these probes express at roughly constant levels for periods as long as months, identified cells from a single organism can be repeatedly imaged over a long period of time (Mank et al., 2008; Tian et al., 2009; Kuhn et al., 2012; Margolis et al., 2012). As an example, Mank et al. (2008) showed that layer 2/3 cells in primary visual cortex of mice expressing TN-XXL exhibit stable tuning to orientation when imaged over the course of weeks. Another study, in contrast, found that at a cellular level, hippocampal place cell response to place fields is highly variable over a similar period of time. Despite the fact that most cells did not retain consistent mapping with respect to the place fields, at the population level an accurate spatial map was preserved (Ziv et al., 2013).

Genetically encoded calcium indicators has also been used to probe the dynamic changes of neural ensembles in response to learning. For example, it was found that as mice were trained to perform a sensorimotor task, preferred stimuli of individual L2/3 neurons expressing GCaMPs in vibrissal motor cortex shifted over time. At the population level, however, activity became increasingly stereotyped and time-locked to the stimulus and motor outputs (Huber et al., 2012). These results suggest that L2/3 neurons in mice motor cortex integrate sensory input to task-related motor programs.

Finally, sensory deprivation has been shown to result in a rapid rescaling of synaptic weights that act to preserve mean levels of neuronal activity around a homeostatically regulated set point via *in vivo* calcium imaging using GCaMPs.(Turrigiano et al., 1998; Bishop and Zito, 2013). In this study the activity of L2/3 and L5 neurons expressing GCAMP3/5 in primary visual cortex of mice was monitored. It was shown that bilateral retinal lesion resulted in a rapid decrease in population activity, followed by recovery to baseline activity levels over the course of the next 24 h (Bishop and Zito, 2013).

## FUTURE PERSPECTIVES

In this review, we have presented a broad overview of the history, current use, and future prospects for the use of GINAs in imaging neural activity in the intact mammalian brain. Through iterative optimization, GINAs—and particularly GECIs—have come to represent an important tool set for systems neuroscientists.

Despite these gains, the power of GINAs in different experimental approaches remains to be fully realized. Of particular importance, optimizing GEVIs to the point that they are widely applicable for *in vivo* imaging is an active area of protein engineering research. Synaptic transmission is another rich area of research that can be better accessed optically through developing novel GINAs that permit probing neurotransmitters, neuropeptides and neuromodulators at the single neuron level as well as at circuit level. For all GINA classes, further development and optimization of color-shifted variants will enhance options for multiplexing optical genetic tools within a single experiment.

The lessons learned and disciplined methods used in optimizing GECIs can be easily adapted for optimizing properties of other GINA classes. As these goals are achieved in the coming years, expect further fundamental contributions to our understanding of brain function to be derived through the use of GINAs in awake, behaving mammals.

## Conflict of Interest Statement

The authors declare that the research was conducted in the absence of any commercial or financial relationships that could be construed as a potential conflict of interest.

## Footnotes

1 https://brainwindows.wordpress.com/

2 http://www.openoptogenetics.org/

3 http://www.addgene.org

4 http://www.med.upenn.edu/gtp/vectorcore/

5 http://www.med.unc.edu/genetherapy/vectorcore

6 http://jaxmice.jax.org

7 http://www.zebrafish.org
